# Assessing the effects of a mixed *Eimeria* spp. challenge on performance, intestinal integrity, and the gut microbiome of broiler chickens

**DOI:** 10.3389/fvets.2023.1224647

**Published:** 2023-08-17

**Authors:** Danielle Graham, Victor M. Petrone-Garcia, Xochitl Hernandez-Velasco, Makenly E. Coles, Marco A. Juarez-Estrada, Juan D. Latorre, Jianmin Chai, Stephanie Shouse, Jiangchao Zhao, Aaron J. Forga, Roberto Senas-Cuesta, Lauren Laverty, Kristen Martin, Carolina Trujillo-Peralta, Ileana Loeza, Latasha S. Gray, Billy M. Hargis, Guillermo Tellez-Isaias

**Affiliations:** ^1^Division of Agriculture, Department of Poultry Science, University of Arkansas, Fayetteville, AR, United States; ^2^College of Higher Studies Cuautitlan, National Autonomous University of Mexico (UNAM), Cuautitlan Izcalli, Mexico; ^3^Department of Medicine and Zootechnics of Birds, College of Veterinary Medicine and Zootechnics (UNAM), Mexico City, Mexico; ^4^School of Life Science and Engineering, Foshan University, Foshan, China; ^5^Division of Agriculture, Department of Animal Science, University of Arkansas, Fayetteville, AR, United States

**Keywords:** coccidiosis, chickens, intestinal permeability, performance, challenge model

## Abstract

A mixed *Eimeria* spp. challenge model was designed to assess the effects of challenge on broiler chicken performance, intestinal integrity, and the gut microbiome for future use to evaluate alternative strategies for controlling coccidiosis in broiler chickens. The experimental design involved broiler chickens divided into two groups: a control group (uninfected) and a positive control group, infected with *Eimeria acervulina* (EA), *Eimeria maxima* (EM), and *Eimeria tenella* (ET). At day-of-hatch, 240 off-sex male broiler chicks were randomized and allocated to one of two treatment groups. The treatment groups included: (1) Non-challenged (NC, *n* = 5 replicate pens); and (2) challenged control (PC, *n* = 7 replicate pens) with 20 chickens/pen. Pen weights were recorded at d0, d16, d31, d42, and d52 to determine average body weight (BW) and (BWG). Feed intake was measured at d16, d31, d42, and d52 to calculate feed conversion ratio (FCR). Four diet phases included a starter d0–16, grower d16–31, finisher d31–42, and withdrawal d42–52 diet. At d18, chickens were orally challenged with 200 EA, 3,000 EM, and 500 ET sporulated oocysts/chicken. At d24 (6-day post-challenge) and d37 (19-day post-challenge), intestinal lesion scores were recorded. Additionally, at d24, FITC-d was used as a biomarker to evaluate intestinal permeability and ileal tissue sections were collected for histopathology and gene expression of tight junction proteins. Ileal and cecal contents were also collected to assess the impact of challenge on the microbiome. BWG and FCR from d16–31 was significantly (*p* < 0.05) reduced in PC compared to NC. At d24, intestinal lesion scores were markedly higher in the PC compared to the NC. Intestinal permeability was significantly increased in the PC group based on serum FITC-d levels. Cadherin 1 (CDH1), calprotectin (CALPR), and connexin 45 (Cx45) expression was also upregulated in the ileum of the PC group at d24 (6-day post-challenge) while villin 1 (VIL1) was downregulated in the ileum of the PC group. Additionally, *Clostridium perfringens* (ASV1) was enriched in the cecal content of the PC group. This model could be used to assess the effect of alternative coccidiosis control methods during the post-challenge with EA, EM, and ET.

## 1. Introduction

Coccidiosis, a parasitic disease caused by protozoan parasites of the genus *Eimeria*, is a significant health concern in the poultry industry. This disease affects chickens worldwide, leading to substantial economic losses and posing challenges to poultry producers (1). The economic impact of coccidiosis is multifaceted, encompassing direct costs associated with mortality, decreased productivity, and increased medication expenses, as well as indirect costs related to reduced feed conversion efficiency and impaired flock performance (2, 3). Moreover, the shift away from antibiotics for chicken coccidia control has been driven by several factors, including concerns over antibiotic resistance, regulatory changes, and the need for sustainable farming practices (4). To address this concern, there has been a global push to reduce the use of antibiotics in livestock production, including the poultry industry (5). Already, regulatory bodies in many countries have implemented restrictions on antibiotics in animal feed, including those commonly used for coccidia control. These regulations have encouraged the development and adoption of alternative strategies for coccidia control (6). The purpose of the present study is to evaluate the effect of a mixed *Eimeria* spp. challenge model on performance, intestinal integrity, and the gut microbiome of broiler chickens for future application assessing potential intervention strategies to control coccidiosis. This study focused on different aspects such as exploring additional parameters and providing unique insights into the mechanisms of coccidiosis. Our intention was to develop a comprehensive model that can be used to evaluate the effectiveness of different non-drug-based alternatives for controlling coccidiosis in broiler chickens.

## 2. Materials and methods

### 2.1. Experimental design

At day-of-hatch, 240 off-sex male broiler chicks were randomized and allocated to one of two treatment groups. The treatment groups included: (1) Non-challenged (NC, *n* = 5 replicate pens); and (2) challenged control (PC, *n* = 7 replicate pens) with 20 chickens/pen. Pen weights were recorded at d0, d16, d31, d42, and d52 to determine average body weight (BW) and body weight gain (BWG). Feed intake was measured at d16, d31, d42, and d52 to calculate the feed conversion ratio. Four diet phases included a starter d0–16, grower d16–31, finisher d31–42, and withdrawal d42–52 diet. The experimental diets were formulated to approximate the nutritional requirements of broiler chickens as recommended by the NRC (7) and adjusted to the breeder’s recommendations (8). At d18, chickens were orally challenged with 200 *Eimeria acervulina* (EA), 3,000 *Eimeria maxima* (EM), and 500 *Eimeria tenella* (ET) sporulated oocysts/chicken. At d24 (6-day post-challenge), intestinal lesion scores based on the method described by Johnson and Reid (9) were recorded for four chickens/pen (*n* = 20 for NC; *n* = 28 for PC). The entirety of the gastrointestinal tract was scored with scores being specifically assigned to the duodenal, jejunal/ileal, or cecal sections of the intestine. Additionally, on the same day, four random chickens per pen were selected and orally gavaged with 8.32 mg/kg of body weight of fluorescein isothiocyanate-dextran (FITC-d, MW 3–5 KDa; Sigma-Aldrich Co). One hour after FITC-d administration, chickens were humanely euthanized by CO_2_ inhalation. Blood samples were collected from the femoral vein and centrifuged (1,000 × *g* for 15 min) to separate the serum. Serum levels of FITC-d were used as a biomarker to evaluate intestinal permeability as described by Baxter et al. (10), and ileal tissue sections were collected to evaluate gene expression of tight junction proteins (*n* = 8/group). Ileal and cecal contents were also collected at d24 for 16S rRNA sequencing and microbiome analysis (*n* = 8/group). At d37 (19-day post-challenge), lesion scores were also recorded for four chickens/pen (*n* = 20 for NC; *n* = 28 for PC) but no additional samples were collected at this time. Animal handling and experimental procedures were approved by the University of Arkansas Division of Agriculture Institutional Animal Care and Use Committee (#21134).

### 2.2. *Eimeria* spp. strains

*Eimeria maxima* M6 and *E*. *tenella* oocysts were donated by Dr. John. R. Barta, University of Guelph, Canada, and wild-type *E*. *acervulina* oocysts were used for the challenge. Single oocyst-derived stocks of *E*. *maxima* M6, a strain recovered from a broiler flock in Florida, United States, in the mid-1990s (11) and was propagated *in vivo* in *Eimeria-*free chickens and sporulated *in vitro* to obtain a challenge stock. The *E*. *acervulina* strain was obtained from broiler chickens in Arkansas, United States and species confirmation was based on oocyst morphology and intestinal pathology. A preliminary dose titration study was conducted to determine the challenge dose for the trial (Table 1). The multi-species challenge dose was selected based on % reduction in body weight gain during the challenge period and lesion scores 6-day post-challenge. A 25% reduction of body weight gain during the challenge period (compared to the non-challenged control) and a lesion score of ~2 is target for subclinical coccidiosis challenge models (12). Thus, based on preliminary dose titration results, chickens in the PC group were orally challenged with 200 EA, 3,000 EM, and 500 ET sporulated oocysts/chicken at d18.

**Table 1:** Preliminary dose titration study conducted to determine the challenge dose for the trial.

Treatment	% BWG reduction post-challenge compared to NC	Duodenal Lesion score	*p* value	Jejunal/ileal Lesion score	*p* value	Cecal Lesion score	*p* value
Non-challenged Control (NC)	0.00	0	-	0	1.00	0	-
Dose 1: Challenged with 60 EA, 4,000 EM, and 250 ET	37.77	0.17 ± 0.09	0.9301	2.83 ± 0.17	<0.0001	1.03 ± 0.21	0.0010
Dose 2: Challenged with 90 EA, 6,000 EM, and 500 ET	34.83	0.50 ± 0.13	0.2244	2.70 ± 0.17	<0.0001	0.90 ± 0.19	0.0049
Dose 3: Challenged with 120 EA, 8,000 EM, and 500 ET	48.06	0.87 ± 0.27	0.0085	3.43 ± 0.13	<0.0001	1.90 ± 0.23	<0.0001
Dose 4: Challenged with 150 EA, 10,000 EM, and 750 ET	50.72	1.23 ± 0.31	<0.0001	3.40 ± 0.13	<0.0001	1.70 ± 0.24	<0.0001

Lesion scores: Means reported as mean ± standard error. ANOVA used to determine significant differences at *p* < 0.05 between the non-challenged control group and each challenged group by intestinal segment scored. Means further separated using Student’s *t-*test.

### 2.3. RNA extraction, reverse transcription, and qPCR

Total RNA was extracted from 50 mg of ileal tissue (*n* = 8/treatment) collected at d24 or 6-day post-challenge. The tissue was homogenized in 1 mL Trizol following the manufacturer’s protocol (Invitrogen, Waltham, MA, United States). RNA was then resuspended in 40 μL nuclease-free water and then treated with DNase 1 (New England Biolabs, Ipswich, MA, United States). RNA was repurified using the Trizol RNA isolation protocol and resuspended in 35 μL nuclease-free water. RNA concentration and purity were determined using a Nanodrop 1000 (Nanodrop Technology, Willmington, DE, United States). To obtain template cDNA for qPCR, 1 μg of RNA was added to PrimeScript RT Master Mix (Takara Bio USA Inc., San Jose, CA, United States) per the manufacturer’s instructions. cDNA was diluted 1:10 with nuclease-free water. Power SYBR Green Master Mix (Life Technologies, Carlsbad, CA, United States) was used for real-time quantitative PCR (Applied Biosystems 7500 Real-Time PCR system). The oligonucleotide primers for adhesion, tight junction, and gap junction genes have been listed in Table 2 and were previously described by Tabler et al. (13). The qPCR conditions were as follows: 50°C for 2 m, 95°C for 10 m, 40 cycles at 95°C for 15 s, and 58–61°C for 1 m (varied by primer). Data were analyzed by the delta–delta Ct method (14) using 18S as reference.

**Table 2:** Primers used for real-time quantitative PCR.

Gene	Forward primer (5′–3′)	Reverse primer (5′–3′)	Product size (bp)
18S	TCCCCTCCCGTTACTTGGAT	GCGCTCGTCGGCATGTA	60
CDH1	GGGAGCGCGTTGCCTACTA	GAGGGCTGCCCAGATCTGA	57
CALPR	GCTGGAGAAAGCCATTGATGTC	CCCCTCCCGTCTCGAGTAC	61
Cx45	TCCACCTTCGTTGGCAAAA	TCAGAACGATCCGAAAGACGAT	58
VIL1	TGCCGGTGCCCACTAAAA	TCGACAGCAGCACGTAGCA	63
ZO-1	GGGAACAACACACGGTGACTCT	AGGATTATCCCTTCCTCCAGATATTG	80
ZO-2	GCAATTGTATCAGTGGGCACAA	CTTAAAACCAGCTTCACGCAACT	69
ZO-3	CAAAGCAAGCCGGACATTTAC	GTCAAAATGCGTCCGGATGTA	63
OCLN	CGCAGATGTCCAGCGGTTA	GTAGGCCTGGCTGCACATG	59
LCN2	TGCAGCTTGCAGGGAGATG	GCTTCTTGTCCTTGAACCAGTTG	69
GJA1	TGGCAGCACCATCTCCAA	GGTGCTCATCGGCGAAGT	56
PATJ	GGATCCAGCAACGTGTCCTATT	GCATCCAGTGGAGTGTCTTTCC	114
JAMA	TCACCTCGGAGACAAAGGAAGT	ACGCAGAGCACGGGATGT	60

CDH1, cadherin 1; CALPR, calprotectin; Cx45, connexin 45; VIL1, villin 1; ZO-1-3, zonula occludens; OCLN, occludin; LCN2, lipocalin 2; GJA1, gap junction protein alpha 1; PATJ, PALS1-associated tight junction protein; and JAMA, junctional adhesion molecule A.

### 2.4. Microbiome

At d24 or 6-day post-challenge, ileal and cecal contents were collected (*n* = 8/treatment). Samples were stored at RT in RNA/DNA shield. Total genomic DNA of ileal and cecal content samples were extracted using the DNeasy Power Lyzer PowerSoil Kit (Qiagen, Germantown, MD, United States) according to the manufacturer’s protocol. The concentration of DNA was measured using a NanoDrop One (Thermo Fisher Scientific, Madison, WI, United States). The extracted DNA was then diluted to 10 ng/μL. The V4 region of the 16S rRNA was amplified using primer sequences (forward: 5′-GTGCCAGCMGCCGCGGTAA-3′ and reverse: 5′-GGACTAC HVGGGTWTCTAAT-3′) attached with gene-specific Illumina adapters for each sample. The PCR products were determined on a 1% agarose gel and then normalized using a commercial normalization plate [SequalPrep Normalization Plate Kit (Invitrogen, Carlsbad, CA, United States)]. All purified PCR amplicons were pooled together to generate a sequencing library. After the concentration and quality of the library were confirmed by KAPA Illumina Library Quantification Kits (Roche, Indianapolis, IN, United States) and an Agilent 2100 Bioanalyzer (Agilent, Santa Clara, CA, United States), the library was sequenced on a MiSeq sequencer (MiSeq Reagent Kit v2, 500 cycles; Illumina, San Diego, CA, United States). To prevent contaminations from reagents, a mock community, ZymoBIOMICS™ Microbial Community Standard (Zymo, Irvine, CA, United States) and negative of DNA extraction and PCR amplification were included in sequencing as well. The sequencing files obtained from the Illumina sequencer were pre-processed, quality filtered (Q > 30), and analyzed using the QIIME2 (2021.4 release) software (15). The Deblur algorithm was used for sequence trimming, denoising, chimera removal, and feature binning at the amplicon sequence variants (ASV) level (16). A naive Bayes classifier was employed for the assignment of all sequences into bacterial taxonomy using the Greengenes (v13_8 clustered at 99% identity) reference database. The raw data are available in the NCBI SRA database with the BioProject ID PRJNA.

### 2.5. Statistics

All data, excluding microbiome data, were analyzed by a one-way ANOVA using the GLM procedure of SAS (17). Means were further separated using Student’s T test with significance at *p <* 0.05. Alpha diversity, including the Shannon Index and the number of Observed ASVs, was compared using a two-tailed Wilcoxon signed-rank test between two groups. Beta diversity based on Bray-Curtis and Jaccard distances was tested using an analysis of similarity (ANOSIM). The outputs of diversity were visualized using the “ggplot2” package in R (v4.1.2). The linear discriminant analysis (LDA) effect size (LEfSe), an analytical tool for discovering and interpreting biomarkers of high-dimensional data, was used to identify the signature bacteria associated with the growth stages and intestinal segments. LDA score>2 was used as a criterion for judging the significant effect size (18). The signature bacteria were visualized in a heat map using the “heatmap” function in R.

## 3. Results

### 3.1. Performance

The results of the evaluation of body weight (BWG), feed intake (FI), and feed conversion ratio (FCR) in broiler chickens in a mixed *Eimeria* spp. challenge model is summarized in Table 3. Significant (*p* < 0.05) differences in average BWG and FCR between NC and PC groups were observed from d16-31, with the NC having markedly higher BWG and lower FCR compared to the PC. There were no significant differences in feed intake observed between the NC and PC groups during any period evaluated (Table 3). There were no significant differences in mortality between the groups (data not shown).

**Table 3:** Evaluation of body weight (BW), body weight gain (BWG), feed intake (FI), and feed conversion ratio (FCR) in broiler chickens in a mixed *Eimeria* spp. challenge model.

Item	Non-challenged	Challenged
BW, g/broiler		
d0	46.71 ± 0.19	47.35 ± 0.25
d16	532.91 ± 26.28	560.24 ± 26.90
d31	1850.22 ± 55.59	1708.35 ± 65.00
d42	3234.69 ± 43.09	3125.15 ± 81.60
d52	4279.19 ± 59.83	4233.74 ± 99.27
BWG, g/broiler		
d0–d16	486.20 ± 26.26	512.89 ± 26.95
d16–d31	1317.31 ± 32.35^a^	1148.12 ± 43.68^b^
d31–d42	1384.46 ± 22.23	1416.80 ± 30.23
d42–d52	1044.50 ± 77.35	1108.59 ± 63.38
d0–d52	4232.48 ± 59.81	4186.39 ± 99.16
FI, g/broiler		
d0–d16	809.96 ± 24.37	835.89 ± 28.47
d16–d31	2117.81 ± 43.80	2030.36 ± 59.33
d31–d42	2556.50 ± 34.02	2650.84 ± 66.22
d42–d52	2142.48 ± 28.68	2153.72 ± 52.93
d0–d52	7626.75 ± 102.32	7670.79 ± 176.30
FCR		
d0–d16	1.67 ± 0.04	1.63 ± 0.04
d16–d31	1.43 ± 0.01^b^	1.61 ± 0.04^a^
d31–d42	1.53 ± 0.03	1.48 ± 0.02
d42–d52	2.10 ± 0.17	1.98 ± 0.11
d0–d52	1.61 ± 0.02	1.64 ± 0.02

Data expressed as mean ± SE. ^a,b^Non-matching superscripts within rows indicates significant difference at *p* < 0.05.

### 3.2. Lesion scores

Table 4 shows the coccidiosis lesion scores at d24 (6-day post-challenge) and d37 (19-day post-challenge). On d24, chickens in the PC group had significantly (*p* < 0.05) higher lesion scores for EA, EM, and ET compared to the NC. Nevertheless, by d37, only significant lesion scores were observed for EM in the PC group compared to non-challenged chickens (Table 4).

**Table 4:** Evaluation of intestinal lesions scores associated with *Eimeria acervulina*, *Eimeria maxima*, or *Eimeria tenella* at d24 (6-day post-challenge) and d37 (19-day post-challenge) in broiler chickens.

Age (days)	Section of intestine	Lesion scores	
		Non-challenged	Challenged^1^
24	Duodenum	0.00 ± 0.00^b^	0.79 ± 0.11^a^
	Jejunum/ileum	0.00 ± 0.00^b^	3.29 ± 0.15^a^
	Cecum	0.00 ± 0.00^b^	1.57 ± 0.23^a^
			
37	Duodenum	0.00 ± 0.00	0.00 ± 0.00
	Jejunum/ileum	0.05 ± 0.05^b^	0.43 ± 0.10^a^
	Cecum	0.00 ± 0.00	0.04 ± 0.04

Data expressed as mean ± SE. ^a,b^Non-matching superscripts within rows indicates significant difference at *p* < 0.05.

1 At d18, PC chickens were orally challenged with 200 EA, 3,000 EM, and 500 ET sporulated oocysts/chicken.

**Figure 1:**
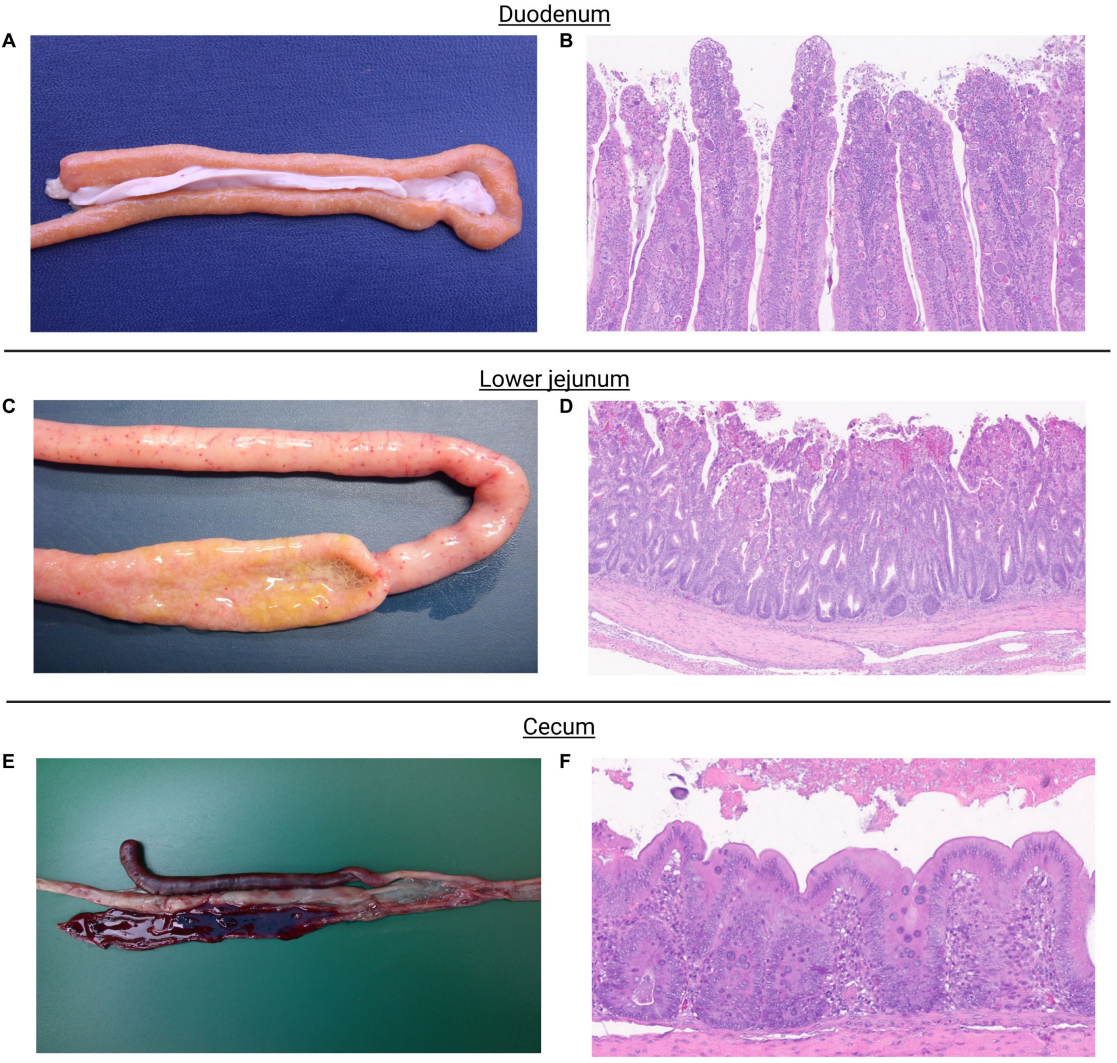
*Eimeria acervulina* primarily affects the duodenum and upper small intestine. Macroscopic lesions include congestion, edema, and thickening of the intestinal mucosa **(A)**. Microscopic examination reveals partial to complete destruction of the duodenal and upper jejunal villi. The damaged villi may exhibit blunting or fusion. The mucosal epithelium may show sloughing or detachment, leading to erosion of the intestinal surface. Endogenous stages of parasite development such as schizonts, microgametes, macrogametes, and immature oocysts can be observed **(B)**. *Eimeria maxima*, the most noticeable macroscopic lesions occur in the small intestine. The affected intestinal segments, particularly the jejunum, appear thickened, congested, and edematous. They may exhibit hemorrhages and appear dark red or black due to the presence of blood. The mucosa may also show a velvety or grainy appearance **(C)**. Microscopic examination shows severe damage to the intestinal mucosa. The villi, which are finger-like projections on the mucosal surface, become shortened and broadened. The epithelial cells lining the villi may be detached, leading to denuded areas. Infiltration of inflammatory cells, such as lymphocytes and heterophils, may also be observed. Endogenous stages of parasite development such as schizonts, microgametes, macrogametes, and immature oocysts can be observed **(D)**. *Eimeria tenella* affects the ceca. Macroscopic lesions in infected chickens involve hemorrhages, congestion, and thickening of the cecal mucosa. The ceca may become distended and contain blood, fibrin, and a characteristic reddish mucoid exudate **(E)**. Microscopic lesions cause severe damage to the cecal mucosa. The cecal epithelium is destroyed, resulting in the formation of ulcers. The base of these ulcers often contains a reddish fibrin necrotic material. Inflammatory infiltrations, including heterophils, lymphocytes, and plasma cells, are commonly observed in the affected areas. Endogenous stages of parasite development, primarily schizonts, can be observed **(F)**.

Figure shows macroscopic and microscopic lesions observed in chickens challenged with the mixed culture of *Eimeria* spp. *Eimeria acervulina* primarily affects the duodenum and upper small intestine. Macroscopic lesions include congestion, oedema, and thickening of the intestinal mucosa (Figure 1A). Microscopic examination reveals partial destruction of the duodenal and upper jejunal villi. The damaged villi may exhibit blunting or fusion. The mucosal epithelium may show sloughing or detachment, leading to erosion of the intestinal surface (Figure 1B).

*Eimeria maxima*, the most noticeable macroscopic lesions, occur in the small intestine. The affected intestinal segments, particularly the jejunum, appear thickened, congested, and edematous. They may exhibit hemorrhages and appear dark red or black due to the presence of blood. The mucosa may also have a velvety or grainy appearance (Figure 1C). Microscopic examination shows severe damage to the intestinal mucosa. The villi, finger-like projections on the mucosal surface, become shortened and broadened. The epithelial cells lining the villi may be detached, leading to denuded areas. Infiltration of inflammatory cells, such as lymphocytes and heterophils, may also be observed (Figure 1D).

*Eimeria tenella* affects the ceca. Macroscopic lesions in infected chickens involve hemorrhages, congestion, and thickening of the cecal mucosa. The ceca may become distended and contain blood, fibrin, and a characteristic reddish mucoid exudate (Figure 1E). Microscopic Lesions cause severe damage to the cecal mucosa. The cecal epithelium is destroyed, resulting in the formation of ulcers. The base of these ulcers often contains a reddish fibrin necrotic material. Inflammatory infiltrations, including heterophils, lymphocytes, and plasma cells, are commonly observed in the affected areas (Figure 1F).

### 3.3. Intestinal permeability and gene expression (tight and gap junctions)

The serum FITC-d and gene expression of tight and gap junctions are summarized in Table 5. Significantly increased serum FITC-d levels were observed for the PC group 6-day post-challenge, which was attributed to the severity of intestinal lesions in the PC group as compared to the NC group. Similarly, significant differences were observed in relative mRNA expressions of cadherin 1 (CDH1), calprotectin (CALPR), connexin 45 (Cx45), and villin 1 (VIL1) between the NC and PC. CDH1, CALPR, and Cx45 were upregulated in the PC group compared to the NC, whereas VIL1 was downregulated in the PC group compared to the NC. However, no significant differences were observed for the other tight or gap junction genes evaluated between both experimental groups (Table 5).

**Table 5:** Evaluation of serum levels of fluorescein isothiocyanate dextran (FITC-d) and relative mRNA expression level of tight and gap junction genes in the ileum of broiler chickens at 6-day post-challenge with mixed *Eimeria* spp.

Item	Non-challenged	Challenged
Serum FITC-d [ng/mL]	34.22 ± 70.43 ^b^	229.50 ± 176.08 ^a^
CDH1	1.00 ± 0.11 ^b^	2.99 ± 0.49 ^a^
CALPR	1.00 ± 0.41 ^b^	255.11 ± 48.75 ^a^
Cx45	1.00 ± 0.17 ^b^	2.91 ± 0.45 ^a^
VIL1	1.00 ± 0.08 ^b^	0.44 ± 0.09 ^a^
ZO-1	1.00 ± 0.10	0.88 ± 0.11
ZO-2	1.00 ± 0.05	1.19 ± 0.16
ZO-3	1.00 ± 0.11	1.43 ± 0.21
OCLN	1.00 ± 0.09	0.88 ± 0.16
LCN2	1.00 ± 0.24	1.36 ± 0.22
GJA1	1.00 ± 0.09	1.26 ± 0.19
PATJ	1.00 ± 0.07	0.88 ± 0.13
JAMA	1.00 ± 0.08	1.10 ± 0.12

^a,b^Non-matching superscripts within rows indicates significant difference at *p* < 0.05. FITC-d, fluorescein isothiocyanate dextran; CDH1, cadherin 1; CALPR, calprotectin; Cx45, connexin 45; VIL1, villin 1; ZO-1-3, zonula occludens; OCLN, occludin; LCN2, lipocalin 2; GJA1, gap junction protein alpha 1; PATJ, PALS1-associated tight junction protein; and JAMA, junctional adhesion molecule A.

### 3.4. Microbiome

**Figure 2:**
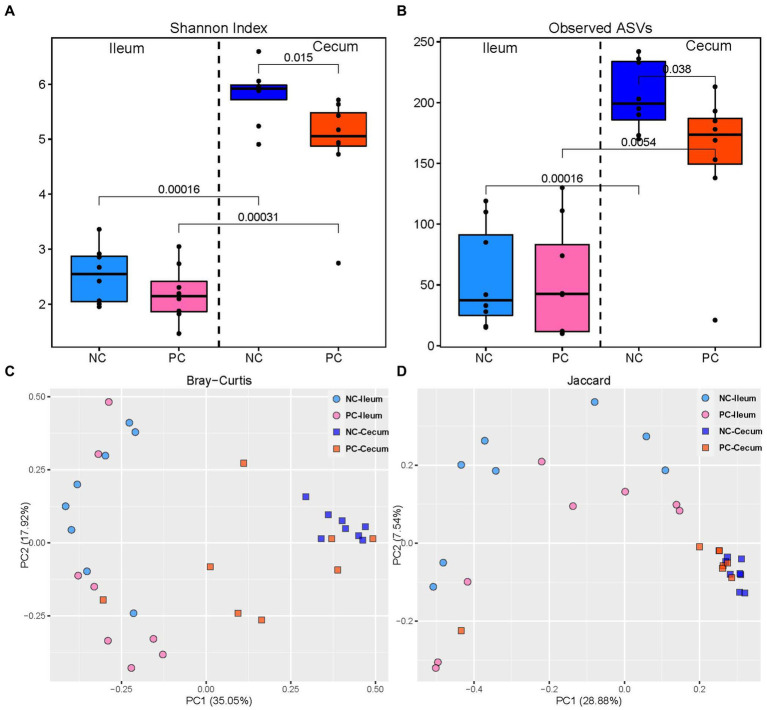
Alpha and beta diversity in ileal and cecal contents; alpha diversity including Shannon Index **(A)** and the number of observed ASVs **(B)** in the ileal and cecal luminal content of broiler chickens. Numbers over bars represent the *p* value. The principal coordinate analysis (PCoA) is based on the Bray—Curtis **(C)** and Jaccard **(D)** distances. Each point represents a unique sample.

The challenge with mixed *Eimeria* spp. did not affect the alpha diversity of the microbial community in the ileal lumen of the PC group compared to the NC group (Figure 2). However, the PC group had a lower alpha diversity [Shannon Index and the number of Observed Amplicon Sequence Variants (ASVs)] in the cecal lumen compared to the NC group. There were no differences in the community structure within the ileal contents of the NC or PC observed on the principal coordinate analysis (PCoA) based on Bray-Curtis and Jaccard distances [Analysis of similarities (ANOSIM): *R* = 0.10, *p* = 0.108; *R* = 0.17, *p* = 0.073; Figure 2]. In contrast, the cecal microbiome of the PC group was distinct compared to NC (ANOSIM: *R* = 0.33, *p* = 0.003; *R* = 0.22, *p* = 0.001). Moreover, higher alpha diversity in the cecal luminal contents was observed in both NC and PC groups, and distinct clusters between cecum and ileum-based Bray-Curtis and Jaccard distances were observed in NC and PC groups (ANOSIM: *R* = 0.99, 0.43, 0.79, 0.33, *p* < 0.05).

**Figure 3:**
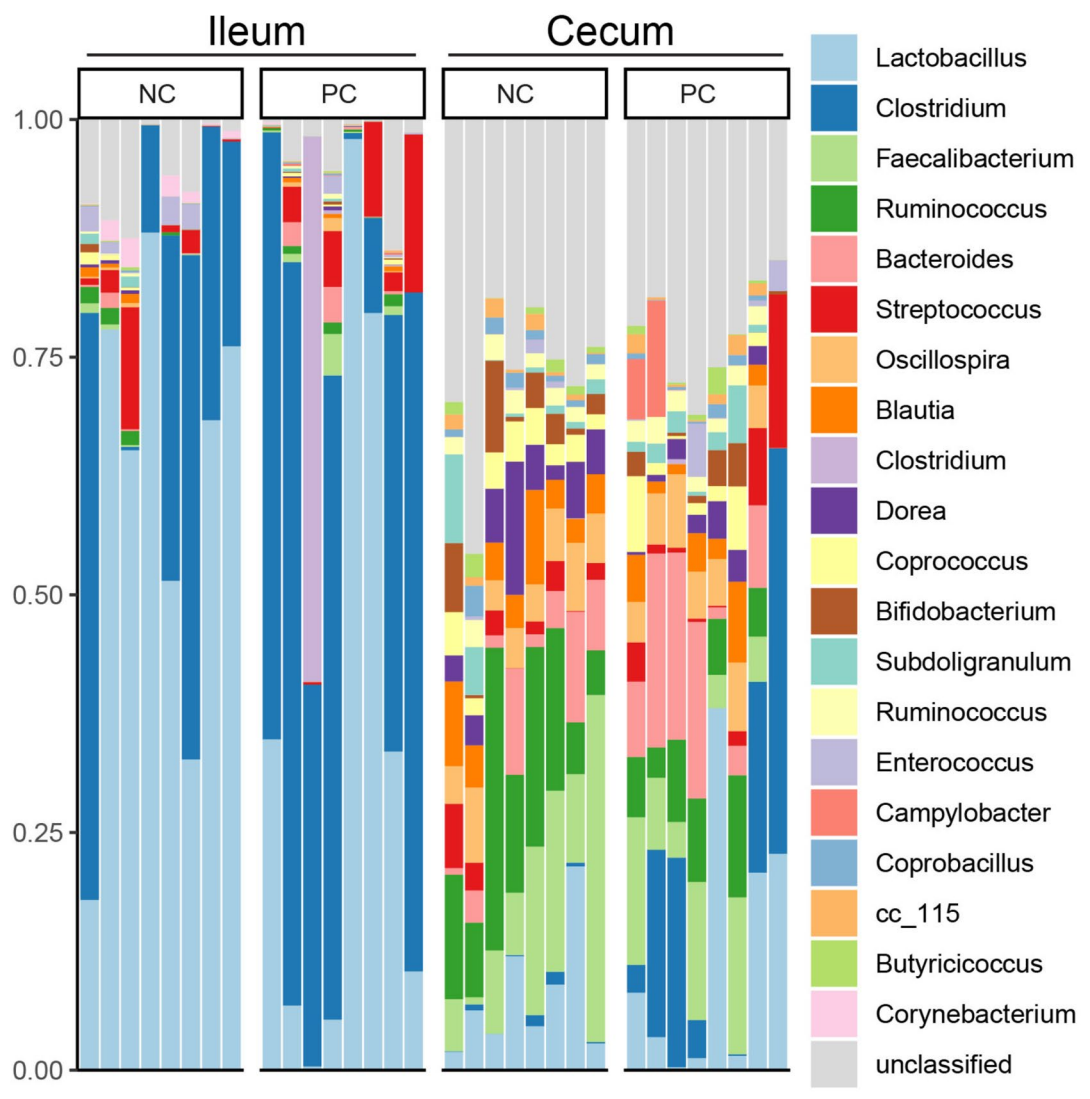
Microbial composition of ileum and cecal content at phylum level. Stacked bar charts demonstrate temporal changes of the top 20 common phyla or genera. Each column represents one sample.

**Figure 4:**
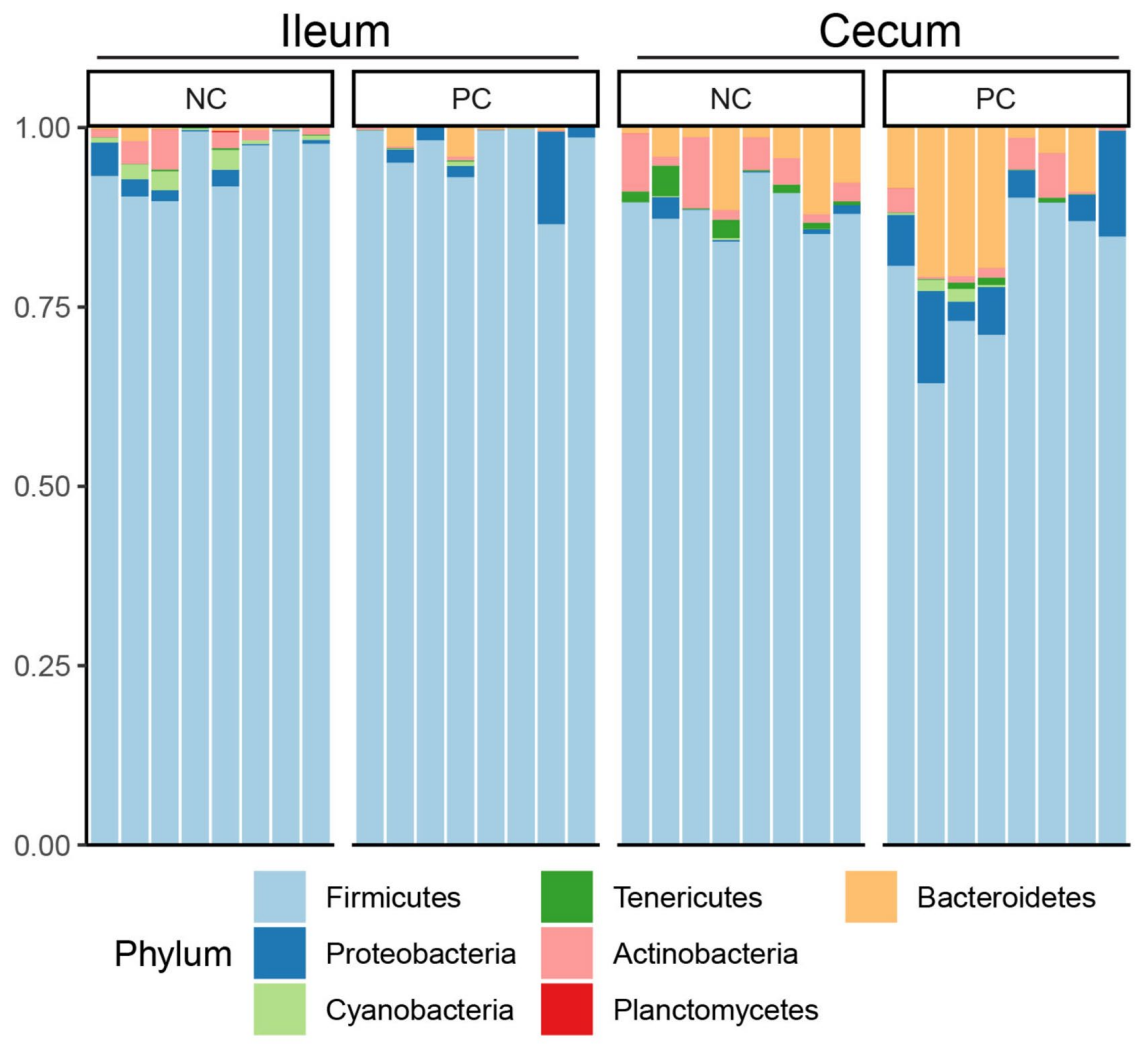
Microbial composition of ileum and cecal content at genus level. Stacked bar charts demonstrate temporal changes of the top 21 common phyla or genera. Each column represents one sample.

At the phylum level, Firmicutes was the dominant bacteria across all samples (Figure 3). Bacteroidetes was also a major phylum in the cecal community. In the ileum, PC had a higher abundance of Proteobacteria (2.43%) compared to NC (1.51%) and a lower abundance of Actinobacteria (0.16%) compared to NC (1.83%). A similar pattern was observed in the cecal community, with Proteobacteria, Actinobacteria, and Bacteroidetes abundance in the NC (0.67, 4.07 and 5.37%) vs. the PC (6.46, 2.16, and 10.41%). At the genus level, the dominant genera across all samples were *Lactobacillus* (28.00%), *Clostridium* (22.02%), *Faecalibacterium* (5.48%), and *Ruminococcus* (5.32%; Figure 4). Although inter-bird variation within treatment groups was observed, treatment effects on the microbiome composition were observed. The average abundance of *Lactobacillus* in the PC (33.58%) was lower than that in the NC (58.99%), while *Clostridium* was higher in the PC (47.12%) compared to the NC (26.72%). For the cecal microbiome, higher abundances of *Faecalibacterium* (12.79%), *Ruminococcus* (13.94%), and *Oscillospira* (5.02%) were observed in the NC, while *Lactobacillus* (11.86%), *Clostridium* (13.78%), *Bacteroides* (9.74%), and *Streptococcus* (3.96%) were higher in the PC.

**Figure 5:**
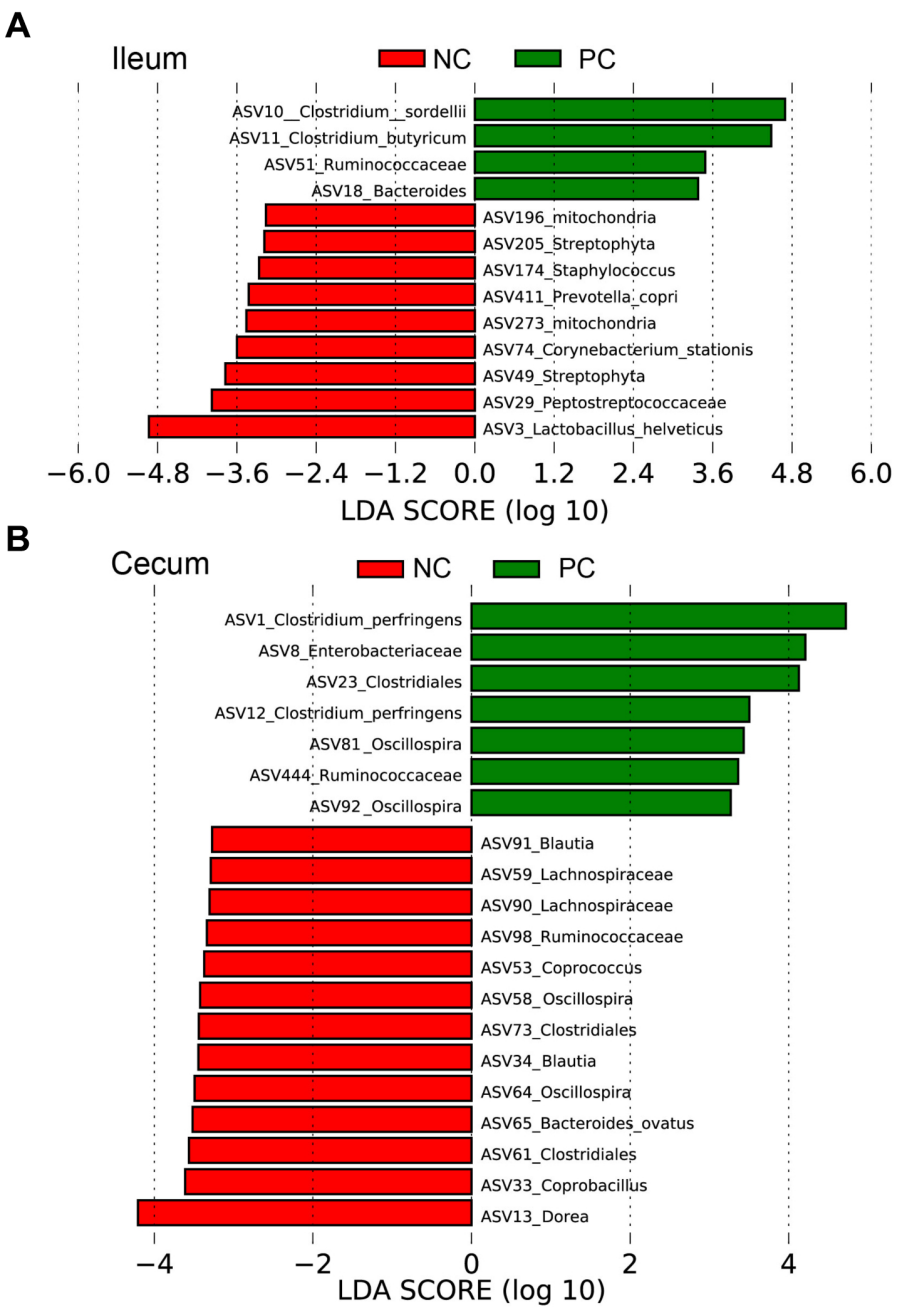
The signature bacteria within each group identified by Linear discriminant analysis Effect Size (LEfSe) to identify bacterial biomarkers for each group at the ASV level. Differentially abundant ASVs between ileum **(A)** and cecum **(B)** contents within the negative control group (NC) and the positive control group (PC). The ASVs in this graph were statistically significant (*p* < 0.05) and had an LDA Score > 2.

Linear discriminant analysis Effect Size (LEfSe) was employed to identify bacterial biomarkers for each group at the ASV level (Figure 5). In the ileum, the signature ASVs for the NC were *Lactobacillus helveticus* (ASV3), *Peptostreptococceceae* unclassified (ASV29), *Streptophyta* (ASV49), and *Corynebacterium stationis* (ASV74), while PC had greater abundances of *Clostridium sordellii* (ASV10), *Clostridium butyricum* (ASV11), *Ruminococceceae* unclassified (ASV51), and *Bacteroides* unclassified (ASV18; Figure 5A). In the cecum, the NC was enriched with *Dorea* (ASV13), *Coprobacillus* (ASV33, ASV58), *Clostridiales* unclassified (ASV61, 73), *Bacteroides ovatus* (ASV65), *Oscillospira* (ASV64), *Coprococcus* (ASV53), and *Lachnospiraceae* unclassified (ASV90, ASV59), while the PC had higher abundances of *Clostridium perfringens* (ASV1, ASV12) and *Enterobacteriaceae* unclassified (ASV8; Figure 5B).

## 4. Discussion

*Eimeria maxima*, *Eimeria acervulina*, and *Eimeria tenella* are three common species that affect chickens (19). Each species produces distinct macroscopic and microscopic lesions in the intestines of infected birds. It is important to note that the severity of lesions may vary depending on the stage of infection, host immunity, and the presence of concurrent infections. FITC-d is a serum biomarker that has been used to assess intestinal permeability in chickens (20). During an *Eimeria* challenge, the parasites invade the intestinal lining, causing damage to the epithelial cells and disrupting the gut homeostasis leading to increased intestinal permeability and allowing molecules, such as FITC-d to leak from the gut lumen into the bloodstream. As a result, serum FITC-d becomes elevated (21). This leakage can occur due to the destruction of epithelial cells, inflammation, and alterations in tight junction proteins that maintain the integrity of the intestinal barrier. This damage can lead to various consequences, including nutrient malabsorption, impaired immune responses, and secondary bacterial infections (22). In the current study, elevated levels of serum FITC-d associated with mixed *Eimeria* spp. infection and marked macroscopic lesion scores indicate the presence of intestinal damage and increased permeability associated with challenge. This was also reflected by performance which was negatively impacted during the d16–31 for PC compared to NC. It is worth noting that while increased levels of serum FITC-d are indicative of intestinal damage during a coccidia challenge, other factors and biomarkers may also be considered to comprehensively evaluate the host response and the progression of the disease.

Adherent junctions are cell adhesion complexes that help maintain the integrity and stability of epithelial tissues by promoting cell–cell adhesion. Cadherin, also known as CDH1 is crucial in regulating cell-to-cell adhesion and is a critical component of adherent junctions in various tissues, including enterocytes lining the intestinal epithelium (23). This adhesive interaction is essential for forming and maintaining the epithelial barrier. In the gut, CDH1-mediated adhesion between enterocytes is crucial for several processes, including regulating paracellular permeability, establishing apical-basal polarity, and maintaining tissue architecture. Loss or dysfunction of CDH1 can lead to compromised barrier function and increased intestinal permeability, which may contribute to various pathological conditions such as inflammation, intestinal injury, and cancer metastasis (24). In the current study, mixed *Eimeria* spp. challenge upregulated CDH1 mRNA expression in the ileum at 6 days post-challenge. Although CDH1 expression in the cecum was not evaluated in the present study, it has been shown to be downregulated post-challenge with *E*. *tenella* (25). The gene regulation of CDH1 can vary in different gut locations, although the core regulatory mechanisms remain largely similar. During an *Eimeria* infection, calprotectin (CALPR) expression can also be upregulated as part of the host immune response (26). CALPR has antimicrobial properties and can inhibit the growth and survival of pathogens, including coccidia (27). CALPR can also modulate the host inflammatory response by regulating the production of pro-inflammatory cytokines and chemokines, preventing excessive inflammation while promoting an effective defense against coccidia (28, 29). In the current study, CALPR expression in the ileum, where *E*. *maxima* invades and develops in the host, was upregulated as a result of challenge. Additionally, in the current study, challenged chickens showed an increase in the expression of Cx45 (connexin 45) gap junction proteins. This upregulation is part of the host’s immune response to the infection and serves several important functions. Connexins are a family of proteins that form gap junctions, allowing for direct cell-to-cell communication and the exchange of small molecules between adjacent cells. Overall, the upregulation of CALPR and Cx45 post-challenge with mixed *Eimeria* spp. in the current study reflects the host’s attempt to mount an effective immune response, including antigen presentation, modulation of cytokines, stress response, immune cell communication, tissue repair, and metabolite exchange. These protein expressions contribute to the overall defense against coccidian parasites and the restoration of tissue integrity.

Gap junctions are specialized intercellular channels formed by connexin proteins, which allow for direct communication and exchange of molecules between adjacent cells. VIL1 is a protein primarily found in the microvilli of intestinal epithelial cells. It has been implicated in forming and maintaining these structures (30). Several mechanisms can contribute to the downregulation of VIL1 gap junction proteins during a coccidia challenge model, including the release of pro-inflammatory cytokines (31–33). In addition, coccidia infection can cause cytoskeletal rearrangements in the infected intestinal epithelial cells, affecting the localization and stability of VIL1 and other junctional proteins, leading to their downregulation (34). Interestingly, chickens challenged with mix *Eimeria* spp. showed downregulation of VIL1 gap junction proteins in the ileum, which may be associated with the reduction in the expression of these proteins in response to coccidia infection. It is important to note that the downregulation of VIL1 during *Eimeria* spp. challenge might be a protective response aimed at limiting parasite replication and spread. By downregulating VIL1 and potentially disrupting gap junction communication, the host may limit the movement of the parasite between infected and uninfected cells. Further research is needed to understand better the specific mechanisms involved and their impact on host–parasite interactions.

In contrast to other studies that have evaluated the expression of other tight junctions (35, 36), there were no significant differences in tight junction expression for junctional adhesion molecule A (JAMA), occludin (OCLN), lipcalin 2 (LCN2), gap junction protein alpha 1 (GJA1), PALS1-associated tight junction protein (PATJ), and zonula occludens 1–3 (ZO-1, ZO-2, and ZO-3) in chickens challenged with *Eimeria maxima*, *Eimeria acervulina*, and *Eimeria tenella* compared with the non-challenged chickens in the current study. Tight junction proteins such as JAMA, OCLN, and ZO1 play a crucial role in maintaining the integrity and function of epithelial cell barriers, including those found in the intestines of chickens. These proteins are involved in forming tight seals between adjacent cells, regulating the passage of molecules and ions, and preventing the entry of pathogens into the underlying tissues (37). However, in the present study, chickens were challenged with *E*. *maxima*, *E*. *acervulina*, and *E*. *tenella*, and the significance of tight junction expression of these proteins appears to be lacking in the ileum at the specific time point evaluated. Despite the crucial role of tight junction proteins in maintaining the integrity of the intestinal barrier, some studies have also suggested that their expression may not be significantly altered during *Eimeria* infection in chickens. One possible explanation for this lack of significance is that the downregulation of tight junction proteins may occur at a post-transcriptional level rather than through changes in gene expression or perhaps sampling at other timepoints would have provided different results. In other words, the expression of JAMA, OCLN, and ZO1 genes may remain relatively constant, but the proteins themselves could be modified or degraded, leading to decreased functional tight junctions. Additionally, it is important to consider that tight junction expression might not be the only factor determining the severity of coccidiosis in chickens.

The host inflammatory response and enteric microbiome can also impact disease progression. The microbiome composition in broiler chickens is influenced by various factors, including genetics. Each chicken’s genetic makeup contributes to the selection of specific microbial communities within the gut and other body sites (38). Genetic variations among individual chickens can impact the diversity, stability, and functionality of their microbiomes. These variations may influence the host’s susceptibility or resistance to diseases, such as *Eimeria* spp. infection (39). Several genetic factors can influence the composition and diversity of the microbiome in broiler chickens. These factors include host genetics related to immune function, mucosal integrity, metabolic pathways, and other physiological traits (40). Genetic variations in immune-related genes, such as pattern recognition receptors (PRRs), cytokines, and antimicrobial peptides, can affect the host’s ability to recognize and respond to *Eimeria* spp. infection. These genetic differences may also influence the composition of the microbiome by altering the availability of nutrients or modulating the immune response toward specific microbial taxa (41). Moreover, the presence of specific commensal bacteria, such as *Lactobacillus* and *Bifidobacterium*, has been associated with improved gut health and resistance to coccidiosis. Genetic variations in the host can shape the microbiome composition and, in turn, impact the host’s susceptibility to *Eimeria* spp. infection (42).

In the present study, challenge with *E*. *acervulina*, *E*. *maxima*, and *E*. *tenella* lowered alpha diversity in the cecal contents but did not affect alpha diversity in the ileal contents suggesting that the presence of *Eimeria* did not significantly alter the overall species richness or evenness in the ileal lumen. Thus, the diversity of microbial species remained relatively stable despite the *Eimeria* infection. There could be several reasons for this observation. The microbial community in the ileal lumen may possess inherent resilience, allowing it to withstand or adapt to the presence of *Eimeria* without experiencing significant alterations in alpha diversity. The microbial community might maintain a dynamic equilibrium despite the temporary disturbance caused by the *Eimeria* infection. Additionally, the immune response of the chickens to *Eimeria* infection could play a role in preserving the alpha diversity of the microbial community. The immune system might modulate the interactions between *Eimeria* and the microbial community, preventing drastic changes in the overall composition and diversity. Furthermore, the experimental conditions and study duration could influence the results. If the study period was relatively short, the effects of *Eimeria* on the microbial community might have yet to fully manifest or reach a point where they could impact alpha diversity. Alternatively, the experimental design may not have been sensitive enough to detect subtle changes in the microbial community. It is important to note that although the alpha diversity remained unaffected, the presence of *Eimeria* species could still lead to other changes in the microbial community, such as alterations in beta diversity (the composition of species between samples) or changes in specific microbial taxa. Therefore, further research is needed to comprehensively understand the interactions between *Eimeria* infection and the microbial community in the ileal lumen of chickens.

In conclusion, administration of a mixed *Eimeria* spp. challenge can be used to evaluate alternative strategies to mitigate the effects of coccidiosis on performance and gut health in broiler chickens. This approach also allows researchers to simulate a more realistic and complex scenario, mimicking the infection with multiple *Eimeria* spp. commonly found in commercial poultry operations. This research contributes to the ongoing efforts to develop sustainable and effective strategies for coccidiosis control, which should be evaluated under controlled, challenged conditions.

## Data Availability

The raw data reads of 16S rRNA gene sequencing of microbiota are deposited in the National Center for Biotechnology Information (NCBI) repository, accession number PRJNA918528.
