# Corrigendum: Assessing the effects of a mixed *Eimeria* spp. challenge on performance, intestinal integrity, and the gut microbiome of broiler chickens

**DOI:** 10.3389/fvets.2024.1548502

**Published:** 2025-01-22

**Authors:** Danielle Graham, Victor M. Petrone-Garcia, Xochitl Hernandez-Velasco, Makenly E. Coles, Marco A. Juarez-Estrada, Juan D. Latorre, Jianmin Chai, Stephanie Shouse, Jiangchao Zhao, Aaron J. Forga, Roberto Senas-Cuesta, Lauren Laverty, Kristen Martin, Carolina Trujillo-Peralta, Ileana Loeza, Latasha S. Gray, Billy M. Hargis, Guillermo Tellez-Isaias

**Affiliations:** ^1^Division of Agriculture, Department of Poultry Science, University of Arkansas, Fayetteville, AR, United States; ^2^College of Higher Studies Cuautitlan, National Autonomous University of Mexico (UNAM), Cuautitlan Izcalli, Mexico; ^3^Department of Medicine and Zootechnics of Birds, College of Veterinary Medicine and Zootechnics (UNAM), Mexico City, Mexico; ^4^School of Life Science and Engineering, Foshan University, Foshan, China; ^5^Division of Agriculture, Department of Animal Science, University of Arkansas, Fayetteville, AR, United States

**Keywords:** coccidiosis, chickens, intestinal permeability, performance, challenge model

In the published article, the **Acknowledgements** statement was erroneously omitted. One of the authors had used generative AI to write their components of the manuscript which was not previously disclosed. The **Acknowledgements** statement appears below.

